# Expression and Clinical Significance of lncRNA NEAT1 in Patients with Spinal Tuberculosis

**DOI:** 10.1155/2022/5748756

**Published:** 2022-04-14

**Authors:** Jianping Zheng, Xiangxin Wang, Jiandang Shi, Jun Tian, Xiuqin Chang, Xiaoping Wang, Qiang Ye

**Affiliations:** ^1^Department of Orthopedic Traumatology, General Hospital of Ningxia Medical University, Yinchuan, Ningxia, China 750004; ^2^Ningxia Medical University, Yinchuan, Ningxia, China 750001; ^3^Department of Spine Surgery, General Hospital of Ningxia Medical University, Yinchuan, Ningxia, China 750004; ^4^Department of Orthopedics, Ningxia Institute for Tuberculosis Control, The Fourth People's Hospital of Ningxia Hui Autonomous Region, Yinchuan, Ningxia, China 750021; ^5^Department of Clinical Laboratory, Ningxia Institute for Tuberculosis Control, The Fourth People's Hospital of Ningxia Hui Autonomous Region, Yinchuan, Ningxia, China 750021; ^6^Tuberculosis Reference Laboratory, Ningxia Institute for Tuberculosis Control, The Fourth People's Hospital of Ningxia Hui Autonomous Region, Yinchuan, Ningxia, China 750021

## Abstract

**Background:**

Spinal tuberculosis (STB) often leads to irreversible neurological injury, resulting in serious social and economic problems. With the emergence of drug resistance, the management becomes even more challenging, given the treatment courses are generally longer for skeletal than pulmonary tuberculosis (PTB). The development and validation of nonsputum biomarkers for diagnosis and tailoring of treatment duration to enable personalized and evidence-based management of such diseases to improve treatment outcomes is being called for globally. Studies have demonstrated that lncRNA NEAT1 was highly expressed in pulmonary tuberculosis (TB) and was related to its progression and recovery. However, the expression and clinical significance of lncRNA NEAT1 in STB remains unclear.

**Methods:**

The relative expression of lncRNA NEAT1 was quantified by relative real-time reverse transcription PCR (RT-PCR). The prognostic value was assessed by receiver-operating characteristic (ROC) curve analysis. Pearson and Spearman correlation coefficient and chi-square test were used to analyze the correlation between the lncRNA NEAT1 expression and the clinical characteristics. Univariate and multivariate logistic regression analyses were used to analyze independent predictors of STB recurrence.

**Results:**

Compared with normal healthy individuals, the expression level of lncRNA NEAT1 in peripheral blood and granulomatous tissues of STB patients was significantly increased. The results of the in vitro Mycobacterium tuberculosis- (*Mtb*-) infected cell model showed that the expression level of lncRNA NEAT1 was significantly upregulated in macrophages infected with *Mtb*, and the difference was statistically significant compared with *Mtb*-uninfected group. The expression level of lncRNA NEAT1 in granulomatous tissue of STB was significantly higher than that in peripheral blood. The expression of lncRNA NEAT1 was related to segments of the lesions, paraspinal abscesses, anti-TB treatment, drug resistance, interleukin-6 (IL-6), C-reactive protein (CRP), and erythrocyte sedimentation rate (ESR). Multivariate analysis results showed that relatively high expression of lncRNA NEAT1_1, the shorter transcript of the NEAT1 gene, was an independent prognostic factor of STB outcome.

**Conclusion:**

LncRNA NEAT1 was highly expressed in peripheral blood mononuclear cells (PBMCs) and granulomatous tissue from patients with STB, as well as in *Mtb*-infected THP-1 cell lines. LncRNA NEAT1 expression was significantly associated with clinical characteristics (paraspinal abscesses, segments of the lesions and anti-TB treatment, IL-6, CRP, and ESR) of patients in STB. Increased expression of lncRNA NEAT1_1 predicted good prognosis of STB and might become a prognostic biomarker for STB.

## 1. Introduction

Spinal has been the most common site of skeletal tuberculosis, and STB amounts for approximately 50-60% of the cases of extrapulmonary musculoskeletal TB (EPTB) [1, 2]. STB often leads to irreversible neurological injury, including paralysis, resulting in serious social and economic problems [3]. Early diagnosis and treatment can improve the prognosis [4]. However, STB usually is insidious in onset and the disease progresses at a slow pace [5]. The diagnostic period, since the onset of symptoms, may vary from 2 weeks to several years [6]. Moreover, the incidence of atypical clinicoradiological presentations of STB is on the rise. When combined with drug-resistant tuberculosis, early diagnosis of STB becomes more difficult because not only is the disease deep-seated, but it is also paucibacillary in nature, making procurement of tissue onerous for pathological and microbiological diagnosis [7]. In addition, the increasing prevalence of immunodeficient survivors (e.g., patients with AIDS, patients on long-term prednisone therapy, organ transplant recipients or patients undergoing chemotherapy for cancer treatment) and the emergence of drug-resistant TB have increased the difficulty of treatment, the recurrence rate of STB and finally resulted in a resurgence of TB [8]. Therefore, it is necessary to explore the development of STB, seek new prognostic biomarkers, discover new therapeutic targets, and ultimately improve the prognosis.

Long noncoding RNAs (lncRNAs) constitute a class of RNAs that are longer than 200 nucleotides. They are not translated into a protein product and instead function as an RNA molecule [9]. LncRNAs are involved in a variety of key biological processes and human diseases, including prostate cancer, lung cancer, papillary thyroid carcinoma, and acute myeloid leukemia [10–15]. LncRNAs have also been reported to act as regulators of gene expression networks and play a significant role in epigenetic regulation of gene expression [16]. A recent study showed that lncRNA-based therapeutics were gaining interest [17]. In addition, increasing evidence indicates that lncRNA is also involved in the immune regulation of the host. Chang et al. reported that tumor or immune cell-derived lncRNAs could regulate immune cells to shape the tumor-suppressive microenvironment [18]. Li et al. found that the expression of PCED1B-AS1 was downregulated in patients with active TB, and PCED1B-AS1 could modulate macrophage apoptosis and autophagy by targeting the miR-155 axis in active TB [19].

Nuclear-enriched abundant transcript 1 (NEAT1) is a highly abundant lncRNA in the mammalian cell nucleus that associates with specific RNA-binding proteins to form NEAT1 ribonucleoproteins (RNPs) [20]. There are two subtypes of lncRNA NEAT1, namely, NEAT1_1 (3.7 kb) and NEAT1_2 (23 kb) [21]. Nitusca et al. reported that NEAT1 could serve as a diagnostic biomarker for prostate cancer [22]. LncRNA NEAT1 is also essential in immune regulation [23]. Zhang et al. reported that the lncRNA Neat1 promoted activation of inflammasomes in mouse macrophages [24]. Activated inflammasomes play an important role in the innate immune defense against tuberculosis and can effectively clear infected *Mtb* through macrophage proptosis and initiating macrophage-mediated proinflammatory response [25, 26]. A recent study showed that LncRNA NEAT1 was highly expressed in peripheral blood mononuclear cells (PBMCs) of TB patients and was associated with the outcome of TB. The decreased expression of NEAT1 weakened the clearance of intracellular *Mtb* by macrophages [27]. However, the expression of the lncRNA NEAT1 in STB has never previously been reported, and its clinical significance remains largely unclear. In the present study, we aim to examine the expression level and clinical significance of lncRNA NEAT1 in STB.

## 2. Materials and Methods

### 2.1. Patients Cohort

This is a descriptive observational study. The study was approved by the Ethical Committee of the General Hospital of Ningxia Medical University (2017090). Informed consent was obtained from all patients and healthy controls involved in this study.

#### 2.1.1. STB Group

A total of 120 patients with STB admitted to our hospital from January 2018 to August 2019 were enrolled. Inclusion criteria were patients diagnosed with STB that required surgical treatment. Exclusion criteria were as follows: (1) patients with uncured PTB; (2) patients with extraspinal tuberculosis; (3) patients with poor general condition and surgical contraindications; (4) patients who were lost to follow-up. The diagnosis was based on clinical symptoms and signs, computed tomography (CT), magnetic resonance imaging (MRI) manifestations, laboratory findings, and pathological evidence of TB. Indications for surgery were as follows: (1) progressive or severe neurological damage; (2) bone destruction of various degrees with severe or developing spinal deformity; (3) persistent back pain due to spinal instability; (4) poor response to medical treatment [6]. All patients were treated with anti-TB chemotherapy and surgical treatment. Diagnosis of drug resistance was made based on drug susceptibility testing (DST), which was performed in culture-positive specimens collected during operation. The cure was defined as no recurrence of TB lesions within 2 years after treatment, maintenance of erythrocyte sedimentation rate (ESR) within the normal range, identification of bone union in lesions by radiographic examination, and disappearance of clinical symptoms for 3 months, and relapse was defined as postoperative recurrence of TB lesions, abscess formation, sinus formation, progressive kyphosis, or internal fixation fractures [28].

#### 2.1.2. Control Group

The control group included 65 healthy individuals who underwent routine annual check-up in our hospital during the same period. All individuals included in the control group met the following criteria: (1) no abnormalities were found on chest x-rays; (2) negative results for the PPD test; (3) no recent contact with TB patients; (4) no history of immunosuppressive drugs intake; (5) no history of hypertension, diabetes, hepatitis, and tumor; (6) no history of autoimmune diseases and acquired immunodeficiency diseases. There was no statistical difference between the two groups in terms of gender and age (*P* > 0.05).

#### 2.1.3. Sample Collection

Morning fasting venous blood was collected in a 5 ml tube with sodium heparin (BD Vacutainer, USA) for anticoagulation on the first day of admission. PBMCs were routinely isolated using the Ficoll lymphocyte separation medium. The TB granulomatous tissues were collected from the lesion of the STB when the operation was performed. The collected tissues were cut into small pieces with a thickness of less than 0.5 cm and immediately placed in RNAwait, stored at 4°C overnight, and then, transferred to -80°C for long-term storage until use.

### 2.2. Reagents

*Mtb* standard strain H37Rv (ATCC 27294) was preserved by the laboratory. THP-1 human macrophages were purchased from the Shanghai Institute of Cell Research, Chinese Academy of Sciences. Phorbol-12-myristate-13-acetate (PMA) and Ficoll lymphocyte separation medium were purchased from Sigma (USA). The RPMI-1640 medium, fetal bovine serum, and phosphate-buffered saline (PBS) were obtained from BI (Israel). Trizol was purchased from Invitrogen (USA). Chloroform, isopropanol, and ethanol were purchased from Damao Chemical Reagent Factory (China). DNase/RNase-free double-distilled water and RNAwait were obtained from Solarbio (China). The PrimeScript reverse transcription reagent kit with gDNA Eraser and TB Green Premix Ex Taq II were purchased from TaKaRa (Japan).

### 2.3. Preparation of H37Rv

*Mtb* strain H37Rv was grown to logarithmic phase in Middlebrook 7H9 broth (BD, USA) supplemented with 10% oleic acidalbumin-dextrose-catalase (OADC), 0.05% Tween-80, and 0.1% glycerol at 37°C for 2–3 weeks. *Mtb* was then counted and stored at −80°C until use. Aliquots of bacterial stocks were thawed at 37°C, mixed vigorously by vertexing, and diluted in RPMI-1640 before infection experiments.

### 2.4. THP-1 Cell Culture and Establishment of the Infection Model

The THP-1 cells were cultivated in RPMI-1640 medium supplemented with 10% FBS, 1% penicillin, and 1% streptomycin at 37°C in a humidified atmosphere with 5% CO_2_. Exponentially growing THP-1 cells were used in subsequent experiments. The cell suspension was adjusted to a concentration of 1 × 10^6^ cells/ml, inoculated in a 6-well plate (2 ml per well), and cultured with PMA (100 ng/ml) for 48 hours. After aggregation and attachment, the cells were infected with *Mtb* standard strain H37Rv at a multiplicity of infection (MOI) of 10 (2 × 10^6^ cells/well, 10 bacilli/cell). Then, THP-1 cells were washed three times with PBS after 4 hours to remove uninfected bacteria and cultured in an incubator at 37°C in the presence of 5% CO_2_. The cells not infected with *Mtb* were set as the control group.

### 2.5. RNA Extraction and cDNA Synthesis

Total RNAs in PBMCs and tissues were extracted and analyzed within 4 hours and 1 week after sample collection, respectively. Total RNA from THP-1 cells was extracted 0, 12, 24, 48, and 96 hours after infection. The extraction of total RNA was done following the manufacturer's instruction provided with Trizol reagent. Briefly, the tissue sample (50-100 mg) was incubated in 1 ml Trizol reagent and homogenized using a homogenizer. Cell samples (THP-1 and PBMCs) were lysed by adding 0.5 ml Trizol reagent per 1 × 10^5^-10^7^ cells. After complete dissociation of the nucleoproteins complex, chloroform was added to the sample according to the principle that 1 ml of Trizol reagent corresponded to 0.2 ml of chloroform. Then, the sample was centrifuged at 4°C (12000 x g, 15 min), and the aqueous phase containing the RNA was transferred to a new tube, followed by an addition of 0.5 ml isopropanol per 1 ml Trizol reagent. The sample was then centrifuged again for 10 min at 12000 x g at 4°C. The supernatant was discarded, and the remaining pellet was resuspended in 1 ml of 75% ethanol per 1 ml Trizol reagent. Finally, RNA pellets were dissolved in DNase/RNase-free double distilled water. The concentration and purity were measured on a nanodrop spectrophotometer (Thermo Scientific, USA) by calculating the ratio of optical density at wavelengths of 260/280 nm. The integrity of the RNA was evaluated by 1% agarose gel electrophoresis, and the RNA integrity number (RIN) was also assayed. The total RNA (1 *μ*g) was then converted into cDNA by using a PrimeScript reverse transcription reagent kit with gDNA Eraser following the manufacturer's protocol. The synthesized cDNA was immediately stored at −80°C until use.

### 2.6. RT-PCR

Quantitative real-time PCR (RT-PCR) was conducted with TB Green Premix Ex Taq II kit according to the manufacturer's instructions. Glyceraldehyde-3-phosphate dehydrogenase (GAPDH) was used as an internal reference [18, 19]. PCR results, recorded as cycle threshold (Ct), were normalized against the internal reference. The relative expression level of lncRNA NEAT1 in PBMCs and tissues of STB patients was measured using normal healthy individuals as a reference, while the relative expression level of lncRNA NEAT1 in *Mtb*-infected THP-1 cells was determined against blank THP-1 cells. Relative gene expression levels were determined by using the 2^−ΔΔct^ method. The primer sequences were as follows: NEAT1_1 forward, 5′-GCCACAACGCAGATTGATGC-3′ and reverse, 5′-AGGCAAACAGG-TGGGTAGGT-3′; NEAT1_2 forward, 5′-TGTCCTGTGAGGGTGGTTGT-3′ and reverse, 5′-GAGGGGCCCATTCAGGA-AAC-3′; GAPDH forward, 5′-CAGGAGGCATTGCTGATGAT-3′ and reverse, 5′-GAAGGCTGGGGCTCATTT-3′. The reaction conditions were set as follows: predenaturation at 95°C for 30 s, 40 cycles of denaturation and annealing at 95°C for 5 s, then left at 59°C for 30 s, and final extension at 59°C for 30 s. Melt curve analysis was conducted to validate amplicon specificity. All experiments were repeated at least three times.

### 2.7. Statistical Analysis

Data analysis was performed using SPSS 26.0 software (IBM Corp., Armonk, New York, USA) and GraphPad Prism 8 (IBM Corp., Armonk, New York, USA). The normality test of the data was evaluated using Kolmogorov-Smirnov and Shapiro-Wilk tests. The normally distributed continuous variable was described as the mean and standard deviation (SD), and nonnormally distributed continuous variables as the median and interquartile range (IQR). Categorical variables were described as frequency and percentage. LncRNA NEAT1 expression levels were categorized as low expression or high expression in relation to the mean value. The difference of lncRNA NEAT1 expression between STB and control groups was compared using an independent sample *t*-test. One-way ANOVA was used for comparison between multiple groups. A chi-square test and Fisher exact test were applied to determine the association between the lncRNA NEAT1 expression and the clinical characteristics. Correlations between continuous variables were evaluated using the Pearson and Spearman correlation coefficient. Univariate and multivariate logistic regression analyses were used to analyze independent predictors of STB recurrence, and odds ratios (OR) with 95% confidence intervals (CI) were reported. The area under the receiver-operating characteristic (ROC) curve was used to evaluate the prognostic value of the LncRNA NEAT1. All statistical tests were 2-tailed, and *P* < 0.05 was considered statistically significant.

## 3. Results

### 3.1. Characteristics of STB Patients and Healthy Individuals

All 120 patients with STB included in this study had complete clinical data. Of these, 63 were male and 57 were female. The age of the patients ranged from 14 to 91 years, and the median age was 54 years. None of the patients had coinfection with HIV. All tissue samples obtained during operation were sent for *Mtb* culture postoperatively. Ninety-one samples from 120 patients (76%) were culture positive for *Mtb*. Nine of the 120 included cases suffered a relapse (7.5%), and the rest were cured. Sixty-five cases included in the control group were all healthy individuals. Of these, 37 were male, and 28 were female. The age of the patients ranged from 20 to 80 years, and the median age was 52 years. The clinical characteristics of STB patients are summarized in Table 1.

### 3.2. The Relative Expression Level of lncRNA NEAT1 in Peripheral Blood and Granulomatous Tissues of STB Patients

**Figure 1:**
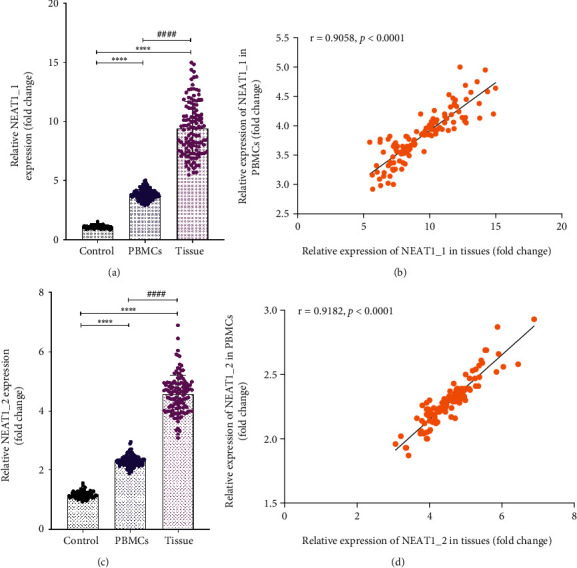
The mean expression level of lncRNA NEAT1 in PBMCs and granulomatous tissues of STB patients. (a) Relative expression level of lncRNA NEAT1_1. (b). Correlation between the expression level of lncRNA NEAT1_1 in peripheral blood and granulomatous tissues. (c) Relative expression level of lncRNA NEAT1_2. (d) Correlation between the expression level of lncRNA NEAT1_2 in peripheral blood and granulomatous tissues. Data were analyzed by using an independent sample *t*-test and Pearson correlation coefficient. *P* value < 0.05 was considered statistically significant. ^∗∗∗∗^*P* < 0.0001 versus the control group (the cohort composed of normal healthy individuals). *^####^P* < 0.0001 versus PBMC group.

In this study, we firstly explored the relative expression level of lncRNA NEAT1 in peripheral blood and granulomatous tissues of STB. The median 260/280 ratio and RIN value of total RNA in peripheral blood were 1.89 (1.84-1.93) and 8.04 (7.38-8.29), while the median 260/280 ratio and RIN value of total RNA in granulomatous tissues were 1.88 (1.85-1.95) and 7.99 (7.33-8.39). Compared with the cohort composed of normal healthy individuals, the relative expression levels of lncRNA NEAT1_1 and NEAT1_2 in PBMCs and granulomatous tissues of STB patients were upregulated, and there was a statistical difference (*P* < 0.05). Then, we further compared the expression levels of lncRNA NEAT1_1 and NEAT1_2 in peripheral blood and granulomatous tissues of patients with STB. The results showed that the relative expression level of lncRNA NEAT1_1 and NEAT1_2 in granulomatous tissues in the lesion was significantly higher than that in peripheral blood, respectively (*P* < 0.05). Finally, we clarified the correlation between the expression level of lncRNA NEAT1 in PBMCs and granulomatous tissues of STB patients. The expression levels of lncRNA NEAT1_1 and NEAT1_2 in tissue samples from patients with STB were positively correlated with their expressions in peripheral blood, respectively (*P* < 0.05) (Figure 1).

### 3.3. Relative Expression Levels of lncRNA NEAT1 at Different Time Points after Infection of Macrophages with *Mtb*

**Figure 2:**
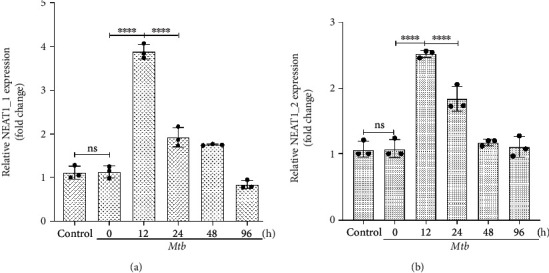
The mean expression level of lncRNA NEAT1 in THP-1 infected with *Mtb*. (a) Relative expression level of lncRNA NEAT1_1. (b) Relative expression level of lncRNA NEAT1_2. *P* values were determined using one-way ANOVA with post hoc LSD correction. *P* value < 0.05 was considered statistically significant. ns: not significant. ^∗∗∗∗^*P* < 0.0001 versus at 24 and 0 hour after infection. *Mtb*: Mycobacterium tuberculosis.

The THP-1 cell line was induced by PMA and differentiated into mature macrophages and then infected with *Mtb* standard strain H37Rv. Total RNA was extracted at 0, 12, 24, 48, and 96 hours after infection, and the expression of RNA at different time points was determined by PCR. The median 260/280 ratio and RIN values of total RNA were 1.87 (1.84-1.92) and 7.9 (7.47-8.59), respectively. RT-PCR results showed that the expressions of lncRNA NEAT1_1 and NEAT1_2 were upregulated at different time points after H37Rv infection in macrophages and reached the peak at 12 hours after infection, which was statistically significant compared with at 0 and 24 hours (*P* < 0.05) (Figure 2).

### 3.4. Association between lncRNA NEAT1 Expression Level and Clinical Characteristics

To further reveal the lncRNA NEAT1 role in STB, we analyzed the correlation between its expression level and the clinical characteristics of STB. The expression levels of lncRNA NEAT1 were divided into high expression and low expression according to its mean value. As shown in Tables 2 and 3, the high expression of lncRNA NEAT1 in peripheral blood and granulomatous tissues was related to greater than or equal to three segments of the lesions, paraspinal abscesses, and anti-TB treatment for less than two weeks, while the low expression of lncRNA NEAT1 is related to newly diagnosed drug resistance. These findings suggest that lncRNA NEAT1 may be involved in the development of STB when the body is infected by MTB. Furthermore, the high expression of lncRNA NEAT1 may also indicate that patients with STB can achieve good outcomes.

### 3.5. The Expression Levels of lncRNA NEAT1 in the Cured and Relapsed Groups and Its Prognostic Value

**Figure 3:**
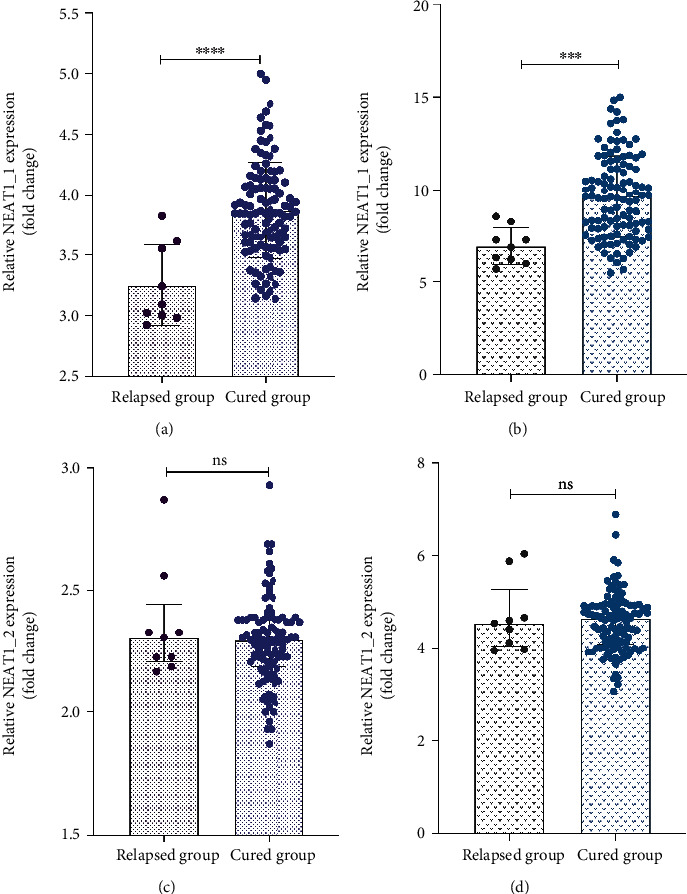
The relative expression level expression level of lncRNA NEAT1 in the cured and relapsed group. (a) Mean expression level of lncRNA NEAT1_1 in peripheral blood of STB. (b) Mean expression level of lncRNA NEAT1_1 in granulomatous tissues of STB. (c) Median expression level of lncRNA NEAT1_2 in peripheral blood of STB. (d) Median expression level of lncRNA NEAT1_1 in granulomatous tissues of STB. Data were analyzed by using independent sample *t*-test and Mann–Whitney *U* test. ns: not significant. ^ns^*P* > 0.05, ^∗∗∗^*P* < 0.001, and ^∗∗∗∗^*P* < 0.0001 versus relapsed group. *P* value < 0.05 was considered statistically significant. STB: spinal tuberculosis.

**Figure 4:**
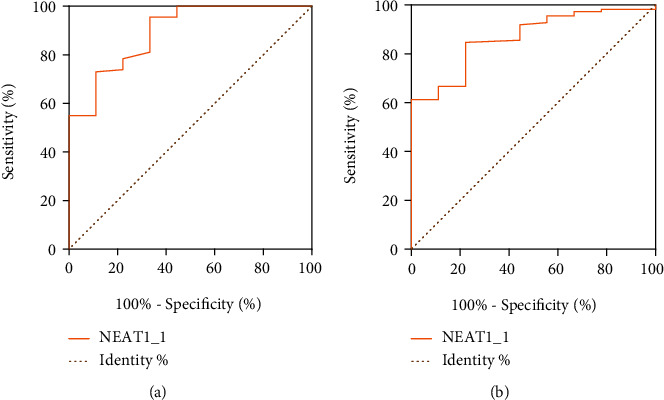
ROC curve displaying the predictive performance of lncRNA NEAT1_1 in the prognosis (cure) of STB patients. (a) Prognostic value of lncRNA NEAT1_1 in peripheral blood of STB. (b) Prognostic value of lncRNA NEAT1_1 in granulomatous tissues of STB; STB: spinal tuberculosis.

All included patients in the STB group were further divided into cured and relapsed group according to the prognosis. Compared with the relapsed group, the expression levels of lncRNA NEAT1_1 in the cured group were upregulated (*P* < 0.05) (Figures 3(a) and 3(b)). However, there was no statistical difference in the expression level of lncRNA NEAT1_2 between the two groups (*P* > 0.05) (Figures 3(c) and 3(d)). The ROC curve showed that the AUC, sensitivity, specificity, and the optimal cut-off for peripheral blood and granulomatous tissues lncRNA NEAT1_1 in the prognosis of STB were 0.893, 96%, 67%, and 3.25 and 0.868, 86%, 78%, and 7.26, respectively (Table 4, Figure 4). Our data demonstrated that STB patients with high lncRNA NEAT1_1 expression showed a good prognosis when compared with those with a low level of lncRNA NEAT1_1.

### 3.6. Correlation between the Expression Level of lncRNA NEAT1 and IL-6, CRP, and ESR in STB

**Figure 5:**
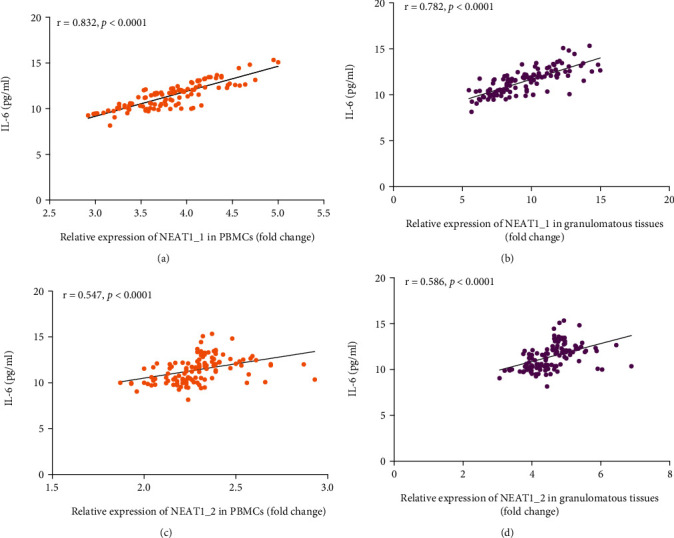
Correlation between the expression level of lncRNA NEAT1 in peripheral blood and granulomatous tissues and IL-6 in peripheral blood serum of STB. Data were analyzed by using Spearman correlation coefficient. *P* value < 0.05 was considered statistically significant. IL-6: interleukin-6; STB: spinal tuberculosis.

**Figure 6:**
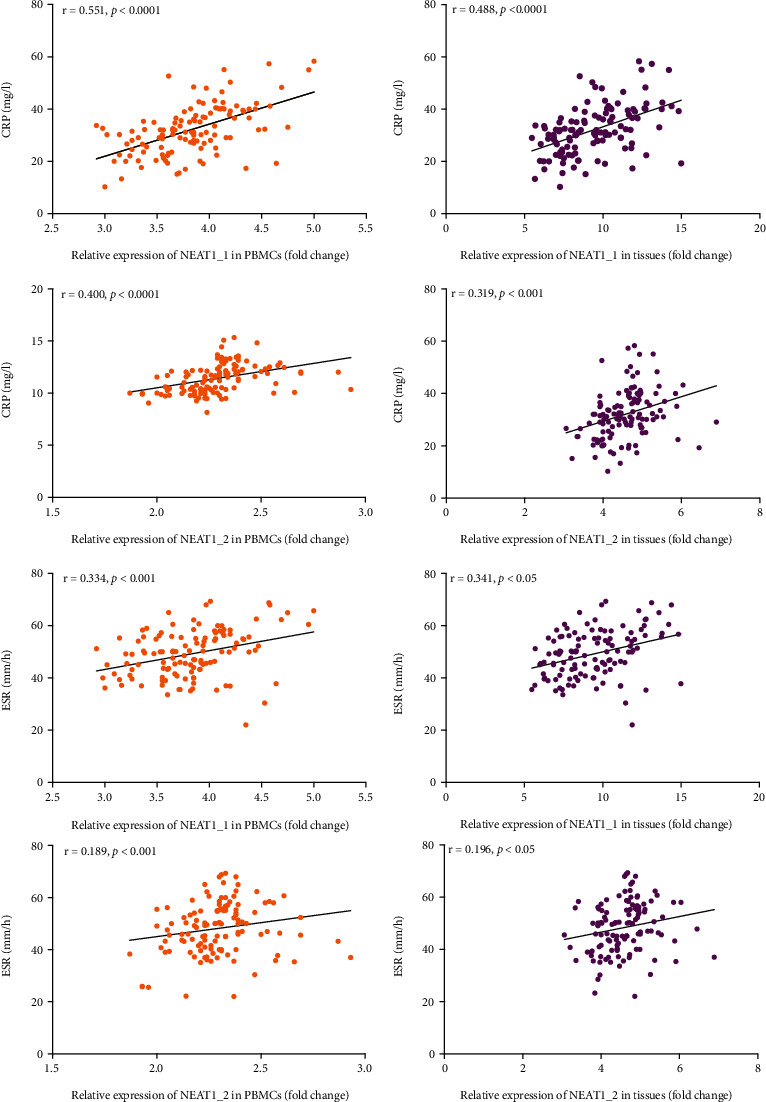
Correlation between the expression level of lncRNA NEAT1 in peripheral blood and granulomatous tissues and CRP and ESR in peripheral blood serum of STB. Data were analyzed by using Pearson correlation coefficient. *P* value < 0.05 was considered statistically significant. CRP: C-reactive protein; ESR: erythrocyte sedimentation rate, STB: spinal tuberculosis.

Increasing studies have shown that lncRNA NEAT1 participates in the host immune regulation. LncRNA NEAT1 could promote the expression of IL-6 to stimulates immune response [29]. When it was silenced, the expression level of IL-6 was downregulated [27]. The results of this study also showed that the high expression of lncRNA NEAT1 was induced by *Mtb* infection and that different treatment outcomes of STB corresponded to different expression levels. Therefore, we inferred that lncRNA NEAT1 also played a significant role in the development of STB through the host immune regulation mechanism. So, we further analyzed the correlation between the expression level of lncRNA NEAT1 and IL-6 in peripheral blood serum. IL-6 in the peripheral blood serum was measured as a laboratory test indicator when the patient was admitted to the hospital. We collected the measured values of IL-6 in 120 patients with STB and analyzed the correlation with the expression level of lncRNA NEAT1. The results suggested that the level of IL-6 was significantly increased, and the expression level of lncRNA NEAT1_1 and NEAT1_2 in peripheral blood and granulomatous tissues was positively correlated with IL-6 (*P* < 0.05), (Figure 5). Given that lncRNA NEAT1 was positively correlated with the expression of the proinflammatory factor IL-6, we further analyzed its correlation with other common clinical inflammatory markers including CRP and ESR. The results showed that the expression level of lncRNA NEAT1_1 and NEAT1_2 in peripheral blood and granulomatous tissues were also positively correlated with CRP and ESR (*P* < 0.05), (Figure 6).

### 3.7. Prognostic Factors of STB Treatment Outcomes

At last, we explored the factors that affect the treatment outcomes of STB. The prognosis was defined as cure or recurrence based on the treatment outcome. Univariate analysis data indicated that sex, anti-TB treatment, drug resistance, and expression level of lncRNA NEAT1_1 in peripheral blood were significantly associated with treatment outcome of STB. Further multivariate analysis results showed that high expression of lncRNA NEAT1_1 in peripheral blood was an independent prognostic factor of STB outcome (Tables 5 and 6).

## 4. Discussion

Accumulating evidence suggested that lncRNAs not only participated in the occurrence and development of many diseases [28] but also participated in the regulation of gene expression and immune response [30‒31]. In addition, research on the use of lncRNA as a prognostic marker is increasing in various tumors including nasopharyngeal carcinoma, gastric cancer, hepatocellular cancer, colorectal cancer, breast cancer, and cervical cancer [32]. Therefore, it is necessary to explore the expression level of lncRNAs in diseases and their clinical significance.

Recent studies showed that lncRNA also plays a regulatory role in the occurrence and development of TB. A study by Sun et al. demonstrated that LncRNA MEG3 affected the biological activity of *Mtb*-infected macrophages by targeting miR-145-5p [33]. Liu et al. reported that the expression of lncRNA SNHG15 was significantly increased in spinal tuberculosis tissues. The downregulation of lncRNA SNHG15 expression could inhibit the secretion of inflammatory cytokines by regulating the RANK/RANKL pathway, thereby regulating osteoclasts [39]. LncRNA NEAT1 was also involved in the regulation of the occurrence and development of PTB [23]. However, there are no reports on the expression and related regulation of lncRNA NEAT1 in STB.

This is the first study on the expression of lncRNA NEAT1 in PBMCs and granulomatous tissues of STB patients and its correlation with clinical characteristics. The results showed that the expression of lncRNA NEAT1_1 and NEAT1_2 in peripheral blood and granulomatous tissues of STB lesions was significantly upregulated compared with healthy individuals, and there was a high positive correlation between them. In addition, the expression levels of lncRNAs NEAT1_1 and NEAT1_2 in THP-1 cells infected with H37Rv were significantly higher than those in the uninfected group. These results suggested that when the body was infected by *Mtb*, the expression of lncRNA NEAT1 was promoted through a certain potential mechanism. Therefore, we further analyzed the association between the expression level of lncRNA NEAT1 and the clinical characteristics of STB. The data showed that the high expression of lncRNA NEAT1 was associated with paraspinal abscesses, segments of the lesions, and duration of anti-TB chemotherapy. The relative expression level of lncRNA NEAT1 was higher in patients with paravertebral abscess, more than two segments and less than two weeks of preoperative antituberculosis chemotherapy. Based on the above results, it could be concluded that lncRNA NEAT1 was indeed involved in the development of STB, and the expression of lncRNA NEAT1 was related to the severity of inflammatory response in patients with STB. Huang et al. found that the expression level of lncRNA NEAT1 in TB patients gradually decreased with the increase of anti-TB chemotherapy time, and the expression level of lncRNA NEAT1 was insignificantly different from that of the healthy control after 6 months of anti-TB chemotherapy [27]. The results of our study also showed that the high expression of lncRNA NEAT1 mainly occurred in patients with severe disease or with a short duration of anti-TB chemotherapy, which was consistent with the current study. However, the underlying reasons for this result still need to be further explored.

Although the results of this study showed that the expression level of lncRNA NEAT1 in THP-1 cells was upregulated at different time points after H37Rv infection, its peak appeared at 12 hours after infection, which was inconsistent with the report of Huang et al. [27]. This result may be attributed to the different MOIs set in the respective experiments. When we infected THP-1 cells with a higher MOI (MOI = 10), it was more favorable for the THP cells to respond to *Mtb* and thus to express lncRNA NEAT1 due to the higher bacterial load, while the MOI used in Huang et al.'s experiment was only half of ours (MOI = 5).

The human body mainly relies on cellular immunity to fight against TB, and macrophages are important immune cells involved in *Mtb* infection [34]. Studies demonstrated that macrophage polarization was involved in the occurrence and development of TB [35]. Wang et al. reported that two types of macrophage polarization (M1 and M2) were present in the tissues and peripheral blood of patients with STB, indicating that macrophages play proinflammatory and anti-inflammatory roles [36]. The results of the study indicated that M1 macrophages functioned at the crux of host defense by eliciting essential proinflammatory responses and bridging innate and adaptive immunities. M1 macrophages mainly released proinflammatory factors such as tumor necrosis factor-*α* (TNF-*α*) and IL-6 and killed *Mtb* infection at an early stage by phagocytosis, presenting as an antigen and initiating an adaptive immune response. Recent studies found that lncRNA NEAT1 was involved in immune regulation and could promote the expression of IL-6 [24, 29]. Therefore, we hypothesized that lncRNA NEAT1 may play an important role in the prognosis of STB through the proinflammatory response mediated by M1 macrophage polarization to release the proinflammatory factor IL-6 to clear *Mtb*. However, this hypothesis still needs to be verified by research. Therefore, we conducted a correlation analysis between the expression level of lncRNA NEAT1 and inflammatory markers including IL-6, CRP, and ESR. We collected the laboratory examination results of IL-6, CRP, and ESR in peripheral blood serum of 120 STB patients when they were admitted to the hospital and then performed a correlation analysis with the expression level of lncRNA NEAT1. The data showed that the level of IL-6, CRP, and ESR in the peripheral blood serum of STB patients was significantly elevated, and their levels were positively correlated with the expression of lncRNA NEAT1, among which IL-6 was most correlated with the expression level of lncRNA NEAT1_1 in peripheral blood and granulomatous tissues (*r* = 0.832, 0.782, respectively). Based on current research and the results of this study, we speculated that the human body can highly express lncRNA NEAT1 after infection with *Mtb*, which may be involved in the proinflammatory response through the polarization of macrophages M1. However, the potential immune regulatory mechanisms need to be further elaborated. All in all, the current results suggested that the lncRNA NEAT1 highly expressed in peripheral blood of patients with STB had good specificity and might be involved in the regulation of the proinflammatory response induced by *Mtb* infection because of its exact positive correlation with inflammatory markers. Higher expression levels might represent a stronger proinflammatory response in patients with STB.

Since lncRNA NEAT1 was related to the proinflammatory response, it also affected the prognosis of STB. We further analyzed the prognosis of STB. The data from univariate and multivariate analyses showed that the high expression of lncRNA NEAT1_1 in peripheral blood was an independent predictor of cure of spinal tuberculosis. Zahrt found that the recurrence and reactivation of TB were likely to be influenced by factors associated with the host's immune status [37]. It could be concluded that the host's immune status was closely related to the treatment outcome of STB. The results of this study also showed that high expression of lncRNA NEAT1 was a favorable factor for the cure of STB. In addition, the high expression of lncRNA NEAT1 mainly appeared in STB patients with more severe disease or shorter treatment time. In short, the host infected with *Mtb* might respond to the upregulation of lncRNA NEAT1, which in turn, induces the body's proinflammatory response and finally intervenes the prognosis of STB. When the host immune function was normal, the more severe the STB infection was, the more likely it was to cause the overexpress of lncRNA NEAT1. The upregulated expression of lncRNANEAT1 was more conducive to induce the pro-inflammatory response to control *Mtb*, which was beneficial to the cure of STB. When STB patients were immunocompromised, the host would not respond to *Mtb*, resulting in the downregulation of lncRNA NEAT1 expression, and ultimately unable to induce sufficient proinflammatory responses to clear *Mtb* in the host. In this case, STB was also prone to relapse. Furthermore, the host immunodeficiency could easily lead to the immune escape of *Mtb* and relapse of STB.

LncRNAs could also be used to predict the prognosis of diseases. Ma et al. found that the 7 identified glycolysis-related lncRNAs constitute a lncRNA signature with prognostic value for clear cell renal cell carcinoma and provide potential therapeutic targets for the treatment of clear cell renal cell carcinoma patients [38]. The ROC curve results showed that lncRNA NEAT1_1 in peripheral blood of STB patients had satisfactory sensitivity, specificity, and AUC in predicting the outcome of STB, which suggested that RNA in peripheral blood might be used to predict prognosis of STB. Therefore, it is concluded that lncRNA NEAT1 may be used for early prognosis assessment of STB. Our findings not only expanded the understanding of lncRNAs as biomarkers for disease prognosis but also provided new perspectives for the prediction of prognosis.

Indeed, this study still had some limitations. First, we did not conduct in-depth research on the molecular mechanism of lncRNA NEAT1 immune regulation, and this will be the focus of our future work. We also realized that our small sample size might be the limit of our multivariate analysis. Furthermore, although the statistical results found that newly drug resistance was associated with low expression of lncRNA NEAT1, we were unable to draw the conclusion about the association between them given the confounding bias introduced by different duration of anti-TB and incomplete positive culture results. At last, the specificity of lncRNA NEAT1_1 as a prognostic biomarker for spinal tuberculosis still needs to be further confirmed by comparing its expression levels in STB and other tissues (such as normal tissues and cancer tissues) in the future.

In conclusion, the results of this study indicated that lncRNA NEAT1 expression was significantly upregulated in peripheral blood and tissues of STB and THP-1 cell lines infected with *Mtb*. Increased expression of lncRNA NEAT1 in peripheral blood of STB patients was significantly associated with paraspinal abscesses, >3 segments of the lesions and 2 weeks of anti-TB treatment. In addition, the expression level of lncRNA NEAT1 was positively correlated with IL-6, CRP, and ESR in peripheral blood serum of STB patients, and increased expression of lncRNA NEAT1_1 was predictive of a good prognosis. Consequently, LncRNA NEAT1 expression was significantly associated with clinical characteristics (paraspinal abscesses, segments of the lesions and anti-TB treatment, IL-6, CRP, and ESR) of patients in STB. Increased expression of lncRNA NEAT1_1 predicted good prognosis of STB and might become a prognostic biomarker for STB.

## Figures and Tables

**Table 1:** Baseline clinical characteristics.

Parameters	Overall cohort (*n* = 120)	Cured (*n* = 111)	Relapse (*n* = 9)	*P* value
Age (years)				
<60	78 (65)	73 (60.8)	5 (4.2)	0.718
≧60	42 (35)	38 (31.7)	4 (3.3)	
Sex				
Male	63 (52.5)	61 (50.8)	2 (1.7)	0.084
Female	57 (47.5)	50 (41.7)	7 (5.8)	
Systemic toxicity symptoms				
Yes	52 (43.3)	47 (39.2)	5 (4.2)	0.499
No	68 (56.7)	64 (53.3)	4 (3.3)	
Back pain				
Yes	90 (75)	85 (70.8)	5 (4.2)	0.225
No	30 (25)	26 (21.7)	4 (3.3)	
Radiating pain				
Yes	67 (55.8)	60 (50.0)	7 (5.8)	0.296
No	53 (44.2)	51 (42.5)	2 (1.7)	
ASIA scale				
A	5 (4.2)	4 (3.3)	1 (0.8)	0.195
B	10 (8.3)	8 (6.7)	2 (1.7)	
C	21 (17.5)	19 (15.8)	2 (1.7)	
D	33 (27.5)	31 (25.8)	2 (1.7)	
E	51 (42.5)	49 (40.8)	2 (1.7)	
Locations of the lesions				
Thoracic segment	54 (45.0)	48 (40.0)	6 (5.0)	0.132
Lumbar segment	60 (50.0)	58 (48.3)	2 (1.7)	
Both	6 (5.0)	5 (4.2)	1 (0.8)	
Segments of the lesions				
2 segments	59 (49.2)	53 (44.2)	6 (5.0)	0.319
≧3 segments	61 (50.8)	58 (48.3)	3 (2.5)	
Paraspinal abscesses				
Yes	62 (51.7)	58 (48.3)	4 (3.3)	0.737
No	58 (48.3)	53 (44.2)	5 (4.2)	
TB history				
Yes	43 (35.8)	39 (32.5)	4 (3.3)	0.720
No	77 (64.2)	72 (60.0)	5 (4.2)	
Anti-TB treatment				
2 weeks	60 (50)	58 (48.3)	2 (1.7)	0.163
>2weeks	60 (50)	53 (44.2)	7 (5.8)	
CRP (mean, SD) (mg/l)	32.09 ± 9.46	32.00 ± 9.45	33.10 ± 10.05	0.963
ESR (mean, SD) (mm/h)	49.19 ± 9.13	49.28 ± 9.21	48.07 ± 8.45	0.666
IL-6 (median, IQR) (pg/ml)	11.58 (10.27-12.26)	10.35 (9.71-12.09)	11.59 (10.31-12.29)	0.215
TSPOT				
Positive	102 (85.0)	96 (80.0)	6 (5.0)	0.133
Negative	18 (15.0)	15 (12.5)	3 (2.5)	
*Mtb* culture				
Positive	78 (65)	70 (58.3)	8 (6.7)	0.158
Negative	42 (35)	41 (34.2)	1 (0.8)	
Drug resistance				
Yes	32 (26.7)	27 (22.5)	5 (4.2)	0.056
No	88 (73.3)	84 (70.0)	4 (3.3)	

The normally and nonnormally distributed continuous variables are indicated as the mean (SD) and median (IQR), respectively. Categorical variables are indicated as frequency and percentage. Differences between groups were evaluated using a 2-sample *t* test (CRP and ESR) or Mann–Whitney *U* test (IL-6). Categorical data were analyzed using the Fisher exact test. TB: tuberculosis; CRP: C-reactive protein; ESR: erythrocyte sedimentation rate; IL-6: interleukin-6; ASIA: American Spinal Injury Association; T-spot: TSPOT: T-Spot®.TB; SD: standard deviation; IQR: interquartile range. *P* value < 0.05 was considered statistically significant.

**Table 2:** The level of lncRNA NEAT1 in peripheral blood of STB patients.

Characteristic	lncRNA NEAT1_1		*P* value	lncRNA NEAT1_2		*P* value
	High	Low		High	Low	
Age (years)			0.687			0.969
<60	42 (35.0)	36 (30.0)		43 (35.8)	35 (29.2)	
≧60	21 (17.5)	21 (17.5)		23 (19.2)	19 (15.8)	
Sex			0.068			0.058
Male	37 (30.8)	26 (21.7)		34 (28.3)	29 (24.2)	
Female	23 (19.2)	34 (28.3)		20 (16.7)	37 (30.8)	
Locations of the lesions			0.751			0.825
Thoracic segment	29 (24.2)	25 (20.8)		24 (20.0)	30 (25.0)	
Lumbar segment	30 (25.0)	30 (25.0)		28 (23.3)	32 (26.7)	
Thoracic and lumbar segment	4 (3.3)	2 (1.7)		2 (1.7)	4 (3.3)	
Segments of the lesions			≤0.001			≤0.001
2 segments	7 (5.8)	52 (43.3)		7 (5.8)	52 (43.3)	
≧3 segments	56 (46.7)	5 (4.2)		47 (39.2)	14 (11.7)	
Anti-TB treatment			≤0.001			≤0.001
2 weeks	51 (42.5)	9 (7.5)		43 (35.8)	17 (14.2)	
>2weeks	12 (10.0)	48 (40.0)		11 (9.2)	49 (40.8)	
Paraspinal abscesses			0.017			0.004
Yes	37 (30.8)	21 (17.5)		34 (28.3)	24 (20.0)	
No	26 (21.7)	36 (30.0)		20 (16.7)	42 (35.0)	
Drug resistance			≤0.001			≤0.001
Yes	4 (3.3)	28 (23.3)		4 (3.3)	28 (23.3)	
No	59 (49.2)	29 (24.2)		50 (41.7)	38 (31.7)	

Categorical variables are indicated as frequency and percentage. The calculated cut-off value for lncRNA NEAT1_1 and lncRNA NEAT1_2 was 3.82 and 2.30, respectively. Relative lncRNA NEAT1 levels below these cut-off values were described as “low,” and those above the value were described as “high.” *P* values were determined using chi-square test and the Fisher exact test. *P* value < 0.05 was considered statistically significant. TB: tuberculosis.

**Table 3:** The level of lncRNA NEAT1 in granulomatous tissues of STB patients.

Characteristic	lncRNA NEAT1_1		*P* value	lncRNA NEAT1_2		*P* value
	High	Low		High	Low	
Age (years)			0.444			0.619
<60	41 (34.2)	37 (30.8)		39 (32.5)	39 (32.5)	
≧60	19 (15.8)	23 (19.2)		23 (19.2)	19 (15.8)	
Sex			0.107			0.104
Male	38 (31.7)	25 (20.8)		37 (30.8)	26 (21.7)	
Female	26 (21.7)	31 (25.8)		25 (20.8)	32 (26.7)	
Locations of the lesions			0.201			0.653
Thoracic segment	30 (25.0)	24 (20.0)		29 (24.2)	25 (20.8)	
Lumbar segment	28 (23.3)	32 (26.7)		29 (24.2)	31 (25.8)	
Thoracic and lumbar segment	5 (4.2)	1 (0.8)		4 (3.3)	2 (1.7)	
Segments of the lesions			≤0.001			≤0.001
2 segments	6 (5.0)	53 (44.2)		3 (2.5)	56 (46.7)	
≧3 segments	54 (45.0)	7 (5.8)		59 (49.2)	2 (1.7)	
Anti-TB treatment			≤0.001			≤0.001
2 weeks	54 (45.0)	6 (5.0)		53 (44.2)	7 (5.8)	
>2weeks	12 (10.0)	48 (40.0)		9 (7.5)	51 (42.5)	
Paraspinal abscesses			0.002			0.003
Yes	39 (32.5)	19 (15.8)		38 (31.7)	20 (16.7)	
No	26 (20.0)	36 (31.7)		24 (20.0)	38 (31.7)	
Drug resistance			≤0.001			≤0.001
Yes	3 (2.5)	29 (24.2)		1 (0.8)	31 (25.8)	
No	57 (47.5)	31 (25.8)		61 (50.8)	27 (22.5)	

Categorical variables are indicated as frequency and percentage. The calculated cut-off value for lncRNA NEAT1_1 and lncRNA NEAT1_2 was 9.42 and 4.57, respectively. Relative lncRNA NEAT1 levels below these cut-off values were described as “low,” and those above the value were described as “high.” *P* values were determined using chi-square test and the Fisher exact test. *P* value < 0.05 was considered statistically significant. TB: tuberculosis.

**Table 4:** Prognostic value of lncRNA NEAT1_1 in peripheral blood and granulomatous tissues.

Diagnostic index	AUC	95% CI	Standard error	Cut-off	Sensitivity (%)	Specificity (%)
NEAT1_1 (blood)	0.893	0.788-0.998	0.054	3.25	96	67
NEAT1_1 (tissue)	0.868	0.772-0.963	0.049	7.26	86	78

Data was presented by AUC, 95% CI, standard error, cut-off, sensitivity, and specificity. The value of NEAT1_1 to predict cure in patients with of STB was tested by ROC analysis. ROC: receiver-operating characteristic; AUC: area under the ROC curve; CI: confidence interval; STB: spinal tuberculosis.

**Table 5:** Univariate analysis of prognostic factors of STB outcome.

Variable	OR (95% CI)	*P* value
Age	1.537 (0.390-6.060)	0.539
Sex	10.122 (1.224-83.688)	0.032^∗^
Locations of the lesions	0.220 (0.046-1.057)	0.059
Segments of the lesions	0.252 (0.050-1.266)	0.094
Anti-TB treatment	9.077 (1.098-75.020)	0.041^∗^
Drug resistance	6.538 (1.528-27.982)	0.055^∗^
Paraspinal abscesses	0.509 (0.121-2.138)	0.356
CRP	1.012 (0.943-1.087)	0.737
ESR	0.985 (0.914-1.062)	0.701
IL-6	0.713 (0.413-1.230)	0.224
LncRNA NEAT1_1 expression	0.053 (0.007-0.433)	0.006∗

*P* values were determined by using univariate logistic regressions analysis. *P* value < 0.05 was considered statistically significant. ^∗^*P* < 0.05. CRP: C-reactive protein; ESR: erythrocyte sedimentation rate; IL-6: interleukin-6; TB: tuberculosis; STB: spinal tuberculosis; OR: odds ratios; CI: confidence intervals.

**Table 6:** Multivariate analysis of prognostic factors of STB outcome.

Variable	OR (95% CI)	*P* value
Sex	8.169 (0.876-76.188)	0.065
Drug resistance	0.117 (0.895-69.647)	0.208
Anti-TB treatment	0.800 (0.058-11.119)	0.868
LncRNA NEAT1_1 expression	0.099 (0.012-0.817)	0.032^∗^

*P* values were determined by using multivariate logistic regressions analysis. *P* value < 0.05 was considered statistically significant. ^∗^*P* < 0.05. TB: tuberculosis; STB: spinal tuberculosis; OR: odds ratios; CI: confidence intervals.

## Data Availability

The data used to support the findings of this study are available from the corresponding author (jiandang-shi@outlook.com, Prof. Dr Shi.) upon reasonable request.
